# Beyond the locus coeruleus: pupil dynamics as a systems-level marker of neuromodulatory influences on hippocampal memory

**DOI:** 10.3389/fnmol.2026.1976085

**Published:** 2026-10-07

**Authors:** Alex Kafkas

**Affiliations:** Andrew Mayes Centre for Cognitive Neuroscience, School of Health Sciences, University of Manchester, Manchester, United Kingdom

**Keywords:** dopamine, episodic memory, hippocampus, locus coeruleus, neuromodulation, norepinephrine, novelty, pupillometry

## Abstract

Dopamine and norepinephrine regulate hippocampal plasticity through molecular, cellular and circuit mechanisms that shape which experiences are encoded and later remembered. Translating these mechanisms into human memory research remains challenging because direct measures of endogenous catecholaminergic activity are difficult to obtain during memory formation. This Perspective argues that pupillometry provides a non-invasive, temporally precise biomarker for bridging this translational gap. Rather than treating pupil dynamics as a simple proxy for locus coeruleus activity or general arousal, I consider whether pupil-linked memory effects can be better understood by relating them to concurrent neuromodulatory, autonomic and hippocampal processes. I further propose that dilation- and constriction-linked encoding profiles may be associated with different physiological configurations across encoding contexts, including conditions that favour model updating or integration with existing knowledge. I outline how multimodal approaches combining pupillometry with functional MRI, neuromelanin-sensitive imaging, pharmacology, magnetic resonance spectroscopy and computational modelling can test these hypotheses directly. Reframing pupillometry in this way provides a scalable route for linking dopamine and norepinephrine mechanisms with human hippocampal memory and for advancing translational research across ageing and disease.

## 1 Introduction

Dopamine (DA) and norepinephrine (NE) are key neuromodulators of hippocampal plasticity, shaping the encoding, consolidation and retrieval of episodic memories. Animal models show that both systems regulate synaptic plasticity through complementary mechanisms, influencing long-term potentiation, memory persistence and the prioritisation of behaviourally relevant experiences. More recent work on reward, novelty, surprise and contextual unexpectedness suggests that catecholaminergic modulation should be understood not as a unitary response to arousal, but as a family of state-dependent mechanisms through which behaviourally significant events are selected for memory (Aston-Jones and Cohen, 2005; Duszkiewicz et al., 2019; Sara, 2009; Schomaker and Meeter, 2015; Shohamy and Adcock, 2010; Takeuchi et al., 2016).

Despite these advances, translating these mechanistic insights into human memory research remains challenging. Direct assessment of catecholaminergic activity in the human brain is inherently difficult. Techniques such as positron emission tomography (PET), neuromelanin-sensitive magnetic resonance imaging (MRI) and pharmacological manipulations have substantially advanced the field (Joshi and Gold, 2020; Murphy et al., 2014), but each provides only a partial window onto the underlying neurobiology. PET offers receptor- or transporter-specific measures but has limited temporal resolution and is costly and invasive. Neuromelanin-sensitive MRI provides valuable information about the structural integrity of catecholaminergic nuclei, particularly the locus coeruleus (LC), yet does not capture their moment-to-moment functional dynamics. Pharmacological approaches can establish causal links between neurotransmitter systems and cognition but often lack anatomical specificity and are influenced by systemic effects. Consequently, obtaining temporally precise, non-invasive measures of endogenous catecholaminergic activity during memory formation remains a major challenge.

A second limitation is the persistent disconnect between mechanistic animal research and human memory studies. Rodent and non-human primate work has provided detailed accounts of how catecholaminergic inputs regulate hippocampal circuits at molecular, cellular and network levels (Duszkiewicz et al., 2019; Lisman and Grace, 2005; Takeuchi et al., 2016). In contrast, human studies typically infer neuromodulatory influences indirectly from behavioural performance or large-scale neuroimaging measures (Joshi and Gold, 2020; Mathôt, 2018; Murphy et al., 2014). As a result, concepts such as phasic LC responses, dopamine-mediated hippocampal plasticity and catecholaminergic gating of memory are often investigated in separate experimental frameworks, limiting opportunities for genuine translational integration.

These challenges highlight the need for biomarkers that can bridge mechanistic neuroscience and human cognition. The central challenge is therefore to connect molecular and cellular accounts of DA and NE modulation with circuit-level dynamics that can be measured during human memory formation. An ideal biomarker would be non-invasive, temporally sensitive, inexpensive, easily combined with neuroimaging and computational modelling, and sufficiently sensitive to capture trial-by-trial fluctuations in neuromodulatory state. Such measures would facilitate direct tests of theories regarding how catecholaminergic systems prioritise information for long-term storage, while also providing translational tools for studying healthy ageing and neurological or psychiatric disorders characterised by altered catecholaminergic function.

Pupillometry has emerged as a promising candidate. Changes in pupil diameter have consistently been linked to activity within the LC-NE system and, more broadly, to behavioural states associated with catecholaminergic modulation, including arousal, surprise, uncertainty and cognitive effort (Joshi and Gold, 2020; Kafkas, 2024; Mathôt, 2018). An expanding body of evidence further demonstrates that pupil dynamics predict successful memory formation and the subjective quality of later recollection (Kafkas and Montaldi, 2011, 2015a; Murphy et al., 2014). Yet these two literatures have largely developed in parallel. Studies of pupil-linked neuromodulation have focused primarily on the physiological processes associated with pupil dynamics, whereas memory studies have focused primarily on their relationship with encoding and retrieval outcomes. This Perspective considers whether integrating these levels of analysis can generate testable hypotheses about how pupil-linked neuromodulatory states relate to hippocampal memory.

In this Perspective, I argue that pupillometry should be reconsidered as a potential translational biomarker linking catecholaminergic mechanisms of hippocampal plasticity with human episodic memory. Importantly, rather than treating pupil dynamics as a narrow proxy for LC activity or as a general index of arousal, I propose that they relate to concurrent variation in neuromodulatory, autonomic and hippocampal processes during memory formation. This argument does not imply that pupil diameter provides transmitter-specific information in isolation; rather, its value lies in its temporal sensitivity and in its ability to be integrated with converging neural measures. This framework provides a basis for unifying molecular, systems and cognitive neuroscience while identifying priorities for future multimodal and translational research. The perspective advanced here is that pupil dynamics should be interpreted not as transmitter-specific readouts, but as temporally precise physiological measures that can be examined alongside neural and behavioural indices of memory encoding and retrieval.

The central claim is developed in three steps. First, pupillometry provides a temporally precise physiological marker of neuromodulatory state during memory encoding and retrieval. Second, I consider whether pupil-linked memory effects may be understood more fully by situating established LC–pupil relationships within the broader hippocampal, autonomic and neuromodulatory context of memory encoding. Third, opposing pupil responses, including both constriction and dilation, may be associated with different physiological states under different encoding conditions.

## 2 Pupillometry as a systems-level biomarker of hippocampal neuromodulation

Understanding how dopamine and norepinephrine regulate human hippocampal memory requires physiological measures that can capture rapid fluctuations in neural state during encoding and retrieval. Pupillometry is attractive in this respect because it is non-invasive, inexpensive, temporally precise and readily combined with neuroimaging, pharmacology and computational modelling. Initially interpreted mainly as a marker of autonomic arousal or cognitive effort, pupil dynamics are now understood to covary with neuromodulatory brain states linked to LC activity, adrenergic and cholinergic signalling, uncertainty, surprise and behavioural relevance (Joshi and Gold, 2020; Mathôt, 2018; Murphy et al., 2014; Reimer et al., 2016). However, pupil size should not be treated as a direct readout of any single transmitter system or nucleus. Pupillary responses emerge through coordinated sympathetic and parasympathetic pathways and are shaped by distributed cortical, subcortical and brainstem circuits (McDougal and Gamlin, 2015; Steinhauer et al., 2004). Their value therefore lies not in transmitter specificity, but in their ability to index the timing and context of integrated neuromodulatory states during cognition. Here, neuromodulatory state refers to a temporally varying physiological configuration characterised by concurrent changes in pupil dynamics, neuromodulatory and autonomic activity, hippocampal processing and behavioural context. The term does not imply that these components are necessarily causally coupled; they may arise through direct interactions, common upstream influences, or both.

A growing memory literature shows that these pupil-linked states are directly relevant to episodic encoding. Encoding-related pupil responses predict later recognition memory, memory strength, associative learning and mnemonic discrimination across a range of paradigms (Kafkas and Montaldi, 2011; Miller and Unsworth, 2019; Naber et al., 2013; Pajkossy et al., 2020; Papesh et al., 2012; Wetzel et al., 2020). Pupil dynamics also distinguish qualitative aspects of memory, including recollection, familiarity, novelty and false recognition (Kafkas et al., 2023; Kafkas and Montaldi, 2012, 2015b). These findings suggest that pupil responses are not merely epiphenomenal indices of effort or arousal, but physiological signatures associated with whether an experience is encoded and subsequently remembered.

Importantly, pupil responses are sensitive to computational variables that are central to hippocampal learning. Novelty, expectation and prediction error all modulate pupil dynamics during encoding, and these effects can predict different subsequent memory outcomes. For example, unexpected novelty is associated with increasing pupil dilation and enhanced later recollection, whereas expected novelty can elicit pupil constriction that also predicts successful memory (Kafkas, 2021). This dissociation suggests that successful encoding is not supported by a single pupil response profile. Rather, different forms of novelty and expectation may be associated with different relative contributions of neuromodulatory and autonomic processes during encoding, through which information is prioritised, integrated or used to update existing models of the environment.

The critical limitation of an LC-centred interpretation is that it explains why pupil dynamics may accompany neuromodulatory engagement, but not how these physiological states become coupled to hippocampal computations that determine memory formation. Recent simultaneous pupillometry-fMRI evidence supports a broader systems-level account. During incidental encoding, encoding-related pupil responses covaried with hippocampal and parahippocampal activity and predicted subsequent recognition memory. Successful encoding was also associated with pupil-related functional interactions between the hippocampus, Edinger-Westphal nucleus, ventral striatum and orbitofrontal cortex (Kafkas et al., 2026). These findings indicate that pupil-linked memory effects covary with activity and functional connectivity across hippocampal, cortical and brainstem regions, extending the neural context in which established LC–pupil relationships can be considered (Figure 1). Whether these associations reflect causal interactions, common upstream influences, or both warrants further investigation. Importantly, these associations do not establish the direction of influence among these systems. One possibility is that hippocampal, neuromodulatory and autonomic signals interact dynamically during encoding; another is that they are jointly modulated by a broader physiological or behavioural state. These accounts are not mutually exclusive. The proposal advanced here is therefore not that hippocampal–midbrain interactions directly generate pupil responses, but that pupil-linked memory effects may be most informative when interpreted within the broader neural and physiological context in which encoding occurs. Distinguishing direct interactions from common upstream influences will require causal and multimodal approaches.

**Figure 1:**
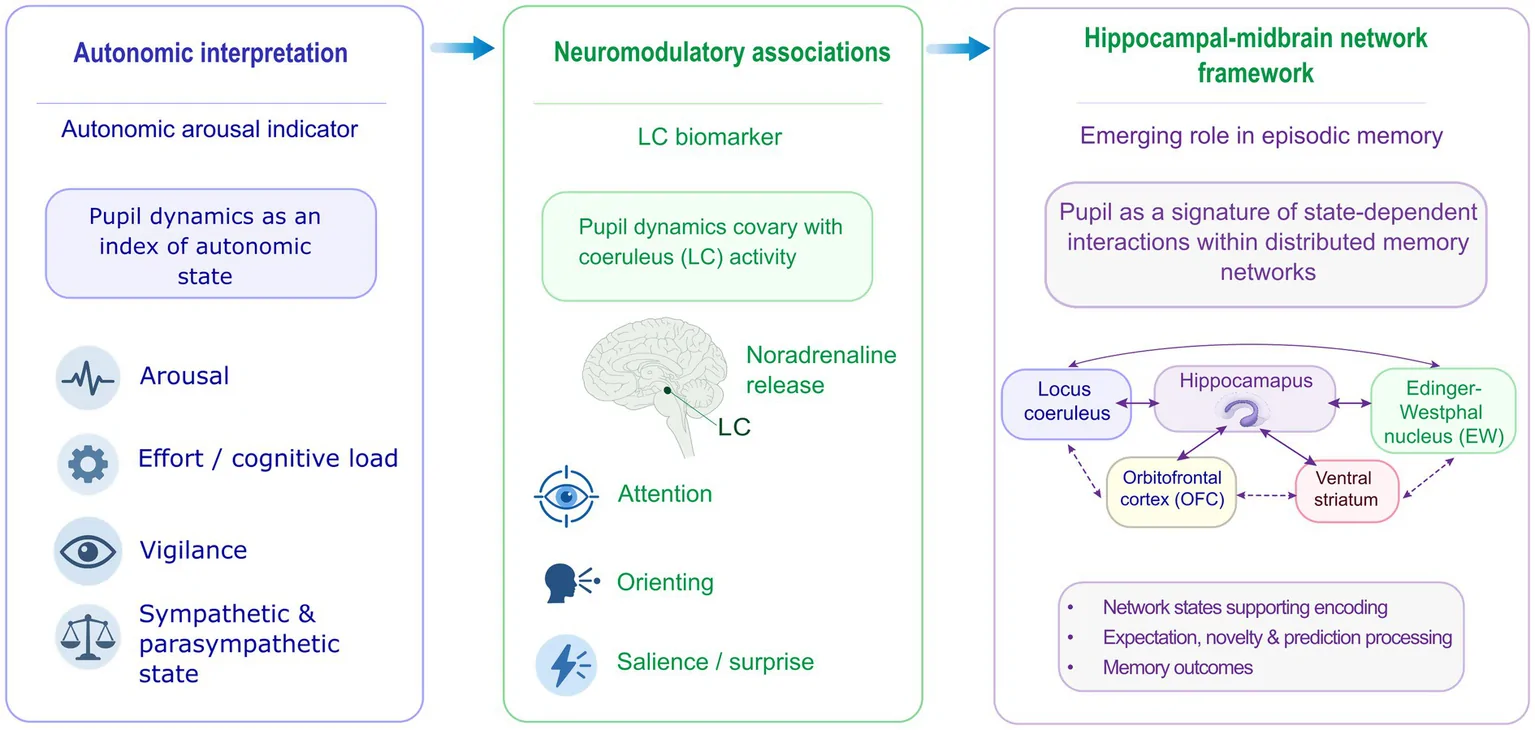
Established interpretations and a proposed systems-level account of pupil dynamics in memory. Rather than indexing locus coeruleus activity alone, pupil dynamics are proposed to reflect coordinated interactions among catecholaminergic nuclei, autonomic brainstem pathways, hippocampal computations and cortical networks. This systems-level signal provides a non-invasive bridge between neuromodulatory mechanisms and human episodic memory.

I therefore propose that pupillometry may be usefully conceptualised as a systems-level marker of hippocampal neuromodulation. In this framework, pupil dynamics are treated as a temporally precise physiological signal that can covary with changes in neuromodulatory and autonomic activity, hippocampal processing and behavioural context during memory encoding. This does not imply that pupillometry can identify dopamine or norepinephrine activity in isolation. Instead, its value lies in providing a temporally precise physiological signal that can be combined with converging neural and computational measures to test how neuromodulatory states shape memory. This perspective therefore shifts the emphasis from treating pupil dynamics as a proxy for LC activity towards examining how trial-by-trial pupil responses relate to concurrent neuromodulatory, autonomic and hippocampal measures during memory encoding.

## 3 State-dependent neuromodulation of memory: reconciling pupil constriction and dilation

One of the most intriguing observations emerging from the pupillometry literature is that successful episodic memory formation is associated with qualitatively different pupil response profiles. While numerous studies report that larger pupil dilations during encoding predict subsequent memory (for reviews see Goldinger and Papesh, 2012; Kafkas, 2024), other work has demonstrated that encoding-related pupil constriction can also predict successful memory under specific experimental conditions (Kafkas, 2021; Kafkas and Montaldi, 2011; Miller and Unsworth, 2019; Naber et al., 2013; Wetzel et al., 2020). At first glance, these findings appear contradictory. However, I propose that they may be associated with different physiological states recruited under different encoding conditions.

Current theories of hippocampal function emphasise that novelty is not unitary. Information may be novel because it is perceptually new, violates a prediction, conflicts with stored knowledge, or becomes behaviourally relevant in context (Frank and Kafkas, 2021; Kafkas and Montaldi, 2018b; Schomaker and Meeter, 2015). These distinctions matter because they are unlikely to recruit identical neuromodulatory mechanisms. Hippocampal-VTA and LC-hippocampal pathways can support different forms of novelty-related learning (Duszkiewicz et al., 2019; Kafkas and Montaldi, 2015a; Lisman and Grace, 2005) and recent high-resolution fMRI work suggests that reward and contextual unexpectedness engage partially separable salience-related networks during encoding (Yi et al., 2025). Successful encoding should therefore not be expected to produce a single physiological pupil profile.

Behavioural evidence supports this distinction. Using an expectation-learning paradigm, Kafkas (2021) demonstrated that expected and unexpected novel stimuli elicited markedly different encoding-related pupil responses despite both leading to successful memory. Unexpected novelty produced increasing pupil dilation during encoding and preferentially enhanced subsequent recollection, whereas expected novelty was associated with pupil constriction that predicted later recognition. Related fMRI evidence shows that surprise can trigger a temporally extended shift towards an adaptive encoding state, supported by hippocampal, midbrain and perceptual-cortical engagement (Frank et al., 2022; Kafkas and Montaldi, 2015a). Together, these findings suggest that different forms of novelty and expectation violation may engage different encoding processes rather than differing only in processing intensity.

Consistent with this view, recent simultaneous pupillometry-fMRI evidence indicates that encoding-related pupil responses are linked to hippocampal and parahippocampal activity and to functional interactions between the hippocampus and midbrain/autonomic regions (Kafkas et al., 2026). For the present argument, the important point is not the involvement of any single region, but that pupil-linked memory effects covary with activity and functional connectivity across hippocampal, cortical and brainstem regions. Therefore, it is hypothesised that pupil constriction and dilation may index different physiological states associated with successful encoding, rather than simply representing opposite ends of a single arousal continuum (Figure 2).

**Figure 2:**
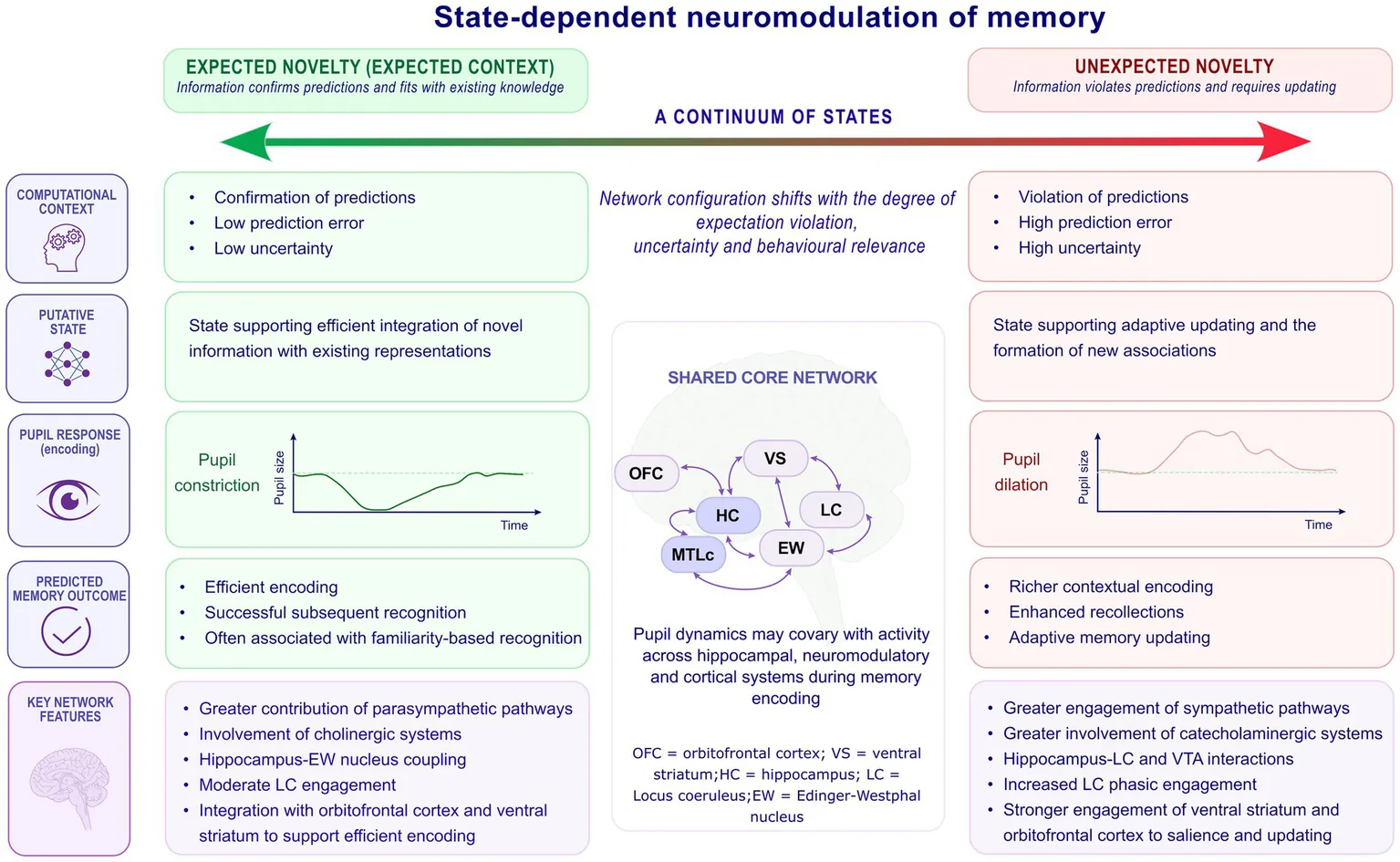
Conceptual model of state-dependent pupil signatures of successful encoding. Expected novelty is proposed to favour integration with existing representations, accompanied by pupil constriction and hippocampal-autonomic brainstem interactions. Unexpected novelty is proposed to favour model updating and recollective encoding, accompanied by pupil dilation and greater engagement of catecholaminergic hippocampal–midbrain interactions. These profiles are presented as hypothesised physiological states associated with different encoding conditions, rather than as established or mutually exclusive neuromodulatory mechanisms. The states are not proposed to be categorical; rather successful encoding may occur along a continuum of physiological configurations whose relative contributions vary with factors such as expectation, novelty and behavioural context.

One hypothesised state supports integration. When novel information is broadly consistent with existing predictions or knowledge, successful encoding may depend on incorporating that information into established representations (Kafkas and Montaldi, 2018a). One possibility is that this state involves a relatively greater contribution of parasympathetic and autonomic brainstem processes. This hypothesis is motivated by the association of expected novelty with pupil constriction and by the observed relationship between encoding-related pupil responses and hippocampal–Edinger–Westphal connectivity (Kafkas et al., 2026). This interpretation is consistent with evidence that expected novelty can predict later memory despite reduced pupil diameter (Kafkas, 2021; Kafkas et al., 2026).

A second hypothesised state supports updating. When events violate expectations or require revision of internal models, successful encoding may rely more heavily on catecholaminergic hippocampal–midbrain interactions. Prediction violations reliably elicit larger pupil dilations, recruit systems involved in adaptive learning, and preferentially enhance recollection (Kafkas, 2021; Kafkas and Montaldi, 2015a). This account is compatible with the GANE model, in which norepinephrine amplifies high-priority representations under salient conditions (Mather et al., 2016), but extends it by linking pupil dilation to hippocampal-midbrain network states during episodic encoding. On this view, dilation during unexpected novelty reflects rapid memory updating rather than simply greater arousal. This proposal concerns relative rather than exclusive neuromodulatory contributions. Indeed, cholinergic and noradrenergic activity can both accompany pupil dilation, but their relationship with pupil dynamics varies according to both timescale and behavioural state. Rapid moment-to-moment fluctuations in pupil dilation more closely track noradrenergic activity, whereas sustained locomotion-related dilation is more closely associated with cholinergic activity (Reimer et al., 2016). This illustrates that a common pupil response need not reflect a single underlying neuromodulatory configuration. Memory formation is likely to depend on continuously changing interactions among cholinergic, noradrenergic and dopaminergic systems, whose relative contributions vary with behavioural context, prior knowledge and uncertainty. Pupil dynamics provide a temporally precise information about these physiological changes, but cannot by themselves identify the contributing neurotransmitter systems or determine whether those systems are causally coupled.

This framework generates a few testable predictions. Manipulating expectation while controlling stimulus novelty should shift encoding-related pupil responses between constriction- and dilation-dominated profiles. If the proposed account is correct, these shifts should be associated with changes extending beyond LC activity alone, including differences in hippocampal–midbrain connectivity. Pharmacological manipulation of cholinergic and catecholaminergic systems should differentially influence these pupil signatures if they reflect distinct neuromodulatory states. An important limitation is that much of the evidence motivating this distinction has emerged from a relatively small set of paradigms. Independent replication and experimental contexts is therefore essential before constriction- and dilation-linked encoding states can be regarded as general features of memory formation. Cross-species work will also be particularly valuable because circuit-level manipulations could determine whether these pupil profiles are causally related to distinct neuromodulatory mechanisms or are parallel consequences of broader physiological states. To conclude, by framing pupil constriction and dilation as signatures of integration-oriented and updating-oriented states, respectively, the model reconciles previously disparate findings and links computational theories of novelty with physiological measures of human memory. Figure 2 summarises this proposal.

## 4 Discussion: testing the framework and future directions

The framework proposed here shifts the emphasis from asking whether pupil responses provide a direct proxy for LC activity towards asking how trial-by-trial pupil dynamics relate to neuromodulatory, autonomic and hippocampal processes during memory encoding. The value of this shift lies in a few testable questions; namely, under which encoding conditions do different pupil profiles occur, which neural and physiological measures covary with them, and how are these profiles related to later memory? A first priority is to move beyond regional activation as the primary explanatory target. Existing neuroimaging studies have identified important correlates of successful encoding in the hippocampus, medial temporal cortex and LC-related regions (Joshi et al., 2016; Lisman and Grace, 2005; Murphy et al., 2014; Takeuchi et al., 2016), but regional effects alone cannot explain how neuromodulatory signals are coordinated with hippocampal plasticity. If pupil dynamics reflect emergent network states, the most informative analyses will be those that relate trial-by-trial pupil fluctuations to interactions among hippocampal, cortical and brainstem regions. In this respect, recent evidence linking encoding-related pupil responses to hippocampal activity and to connectivity between the hippocampus, Edinger-Westphal nucleus, orbitofrontal cortex and ventral striatum provides a useful starting point (Kafkas et al., 2026; see also Kafkas and Montaldi, 2015a). The broader implication is that these relationships should be examined at the network level rather than attributed to one neuromodulatory nucleus by default.

A second priority is methodological integration. No single measure can characterise the complexity of human neuromodulation, so pupillometry should be combined with approaches that provide complementary anatomical, neurochemical and causal information. High-resolution fMRI can relate trial-by-trial pupil fluctuations to hippocampal subfields and network connectivity. Neuromelanin-sensitive MRI can index individual differences in catecholaminergic nuclei, while acknowledging ongoing challenges in LC localisation and measurement (Liu et al., 2017). Pharmacological studies can test whether cholinergic, dopaminergic and noradrenergic manipulations differentially alter constriction- and dilation-linked memory states. Magnetic resonance spectroscopy may provide complementary information about hippocampal or midbrain-adjacent excitatory–inhibitory balance (Kiemes et al., 2021; Stanley et al., 2017), and additional autonomic measures such as heart-rate variability and skin conductance could help dissociate sympathetic and parasympathetic contributions. The key point is not that pupillometry replaces these methods, but that its trial-by-trial temporal resolution can be combined with approaches that provide complementary anatomical, neurochemical and causal information.

A third priority is to connect pupil dynamics with explicit computational accounts of learning. Pupil responses vary with prediction error, uncertainty and expectation (Kafkas, 2021; Pupillo et al., 2023), suggesting that they may index computations that determine when new information is prioritised for storage. Future studies should therefore move beyond simple remembered-versus-forgotten contrasts and estimate latent variables such as surprise, belief updating or uncertainty on a trial-by-trial basis. These variables can then be related simultaneously to pupil responses, hippocampal activity and later memory quality. Such designs would allow the proposed state-dependent framework to be tested more directly by asking whether constriction- and dilation-linked encoding states correspond to different computational routes to memory formation.

The most decisive tests will require causal inference. At present, most evidence linking pupil dynamics to memory is correlational, leaving open whether pupil responses merely accompany successful encoding or index processes that contribute to hippocampal plasticity. The framework proposed here generates falsifiable predictions. Manipulating expectation while holding episodic novelty constant should shift encoding-related pupil responses between constriction- and dilation-dominated profiles. These shifts should be accompanied by changes in hippocampal-midbrain connectivity rather than by LC activation alone. Pharmacological manipulation of cholinergic and catecholaminergic systems should differentially affect these pupil profiles if they reflect distinct neuromodulatory states. Such studies will also be needed to distinguish direct functional coupling among these systems from their parallel modulation by a common physiological state. Finally, the strength of these physiological signatures should predict qualitative differences in memory at retrieval, including the balance between integration with prior knowledge and recollective updating. Conversely, the framework would be weakened if constriction- and dilation-linked memory effects were fully accounted for by a single arousal/LC process, if these pupil profiles were not associated with distinguishable neural or computational states, or if the proposed dissociations failed to generalise across paradigms and independent studies.

The translational relevance of this agenda is substantial. Altered catecholaminergic signalling and hippocampal function are central features of healthy ageing and several neurodegenerative and neuropsychiatric conditions (Dahl et al., 2023; Galgani and Giorgi, 2023; Wu et al., 2025). Because pupillometry is inexpensive, non-invasive and suitable for repeated use, it could help index individual differences in neuromodulatory function during memory formation, especially when combined with structural and functional neuroimaging. Recent evidence that pupil dynamics relate to functional network integrity in ageing (Wu et al., 2025) illustrates this potential. The long-term opportunity is not to use the pupil as a standalone diagnostic marker, but to incorporate pupil dynamics into multimodal profiles of memory function, network integrity and disease progression.

The key opportunity is to use pupillometry not as a proxy for a single neuromodulatory nucleus, but as a temporally precise measure of physiological changes during memory formation that can be related to concurrent neural, autonomic and computational processes. The hypothesis advanced here, therefore, is that constriction- or dilation-linked encoding profiles may be associated with different physiological configurations across encoding contexts, a possibility that requires further testing with converging measures. Thus, testing this proposal will require experimental designs that combine pupillometry with neuroimaging, pharmacology, autonomic physiology and computational modelling. Whether the specific hypotheses advanced here are confirmed or revised, a systems-level view of pupil dynamics may provide a scalable framework for linking molecular and circuit mechanisms of dopamine and norepinephrine modulation with human episodic memory across health, ageing and disease.

## Data Availability

The original contributions presented in the study are included in the article/supplementary material, further inquiries can be directed to the corresponding author.
